# The pathways from disadvantaged socioeconomic status in childhood to edentulism in mid-to-late adulthood over the life-course

**DOI:** 10.1186/s12939-023-01865-y

**Published:** 2023-08-08

**Authors:** Xiaoning Zhang, Shuping Dai, Xue Jiang, Wenhao Huang, Qiong Zhou, Sheng Wang

**Affiliations:** 1 School of Nursing, Hangzhou Normal University, Hangzhou, 311121, China; 2 Zhejiang Philosophy and Social Science Laboratory for Research in Early Development and Childcare, Hangzhou Normal University, Hangzhou, China; 3 School of Marxism, Handong Women’s University, Jinan, China; 4 School of Stomatology, Xuzhou Medical University, Xuzhou, 221004, China; 5 School of Management, Xuzhou Medical University, 209 Tongshan Road, Xuzhou, 221004 China; 6 Department of Nursing, Jiangsu Lianyungang College of Traditional Chinese Medicine, Lianyungang, 222007, China

**Keywords:** Socioeconomic status, Edentulism, Structural equation modeling, Oral health, China

## Abstract

**Background:**

This study aimed to examine the direct and indirect pathways from childhood socioeconomic status (SES) to the prevalence of edentulism in mid-to-late age Chinese individuals using structural equation modeling (SEM).

**Methods:**

This study analyzed data from 17,032 mid- to-late age Chinese individuals in the 2014 and 2015 China Health and Retirement Longitudinal Study (CHARLS). Childhood SES was determined based on the parents’ education and occupation, financial situation of the family, primary residence, food availability, and medical convenience. Adulthood SES was established according to educational achievements of the individuals. Edentulism is defined as the loss of all natural teeth. SEM was used to examine the statistical significance of the association between childhood SES and edentulism, mediated by childhood health, adulthood SES, and adult health.

**Results:**

Childhood SES had significant indirect (*β* = -0.026, *p* < 0.01), and total (*β* = -0.040, *p* < 0.01) effects on edentulism. It was determined that 65% of the total effect of childhood SES on edentulism was indirect, and mainly mediated by adult SES. Also, the goodness-of-fit indices of the best-fitting model were acceptable.

**Conclusion:**

This study revealed that childhood health, adult health and adult SES are mediators that explain the relationship between childhood SES and edentulism. The global attention to alleviate the inequality in edentulism should focus on exploring recommendations and intervention strategies from childhood to adulthood, by considering adult SES, childhood and adult health.

## Background

Oral diseases are a global public health issue, with particular concern about their increasing prevalence over the life course, associated with social and economic changes [1]. They have substantial negative effects on the quality of life of individuals, due to pain, speech difficulties and self-esteem [2] in low- and middle-income countries (LMICs) [3]. The current oral healthcare services are inequitable, and leave disadvantaged people with inadequate basic oral healthcare services [4]. Inequalities in oral health are complicated by individual, social, economic, and environmental determinants that are globally acknowledged across the oral profession [5].

Edentulism is the condition of having lost all natural teeth [6]. Due to its high prevalence [7], the age-standardized prevalence rates of edentulism, according to the Global Burden of Disease (GBD) 2015, was 4.1%, and edentulism was the leading cause of disability-adjusted life years (DALYs) [1]. Edentulism is an irreversible condition, which reflects the lifetime outcome of oral diseases, absence of oral treatment, impaired masticatory function and unhealthy diet [8]. Monitoring edentulism is a major determining factor in the assessment of the performance of the oral healthcare services [9], and a high priority for oral disease prevention efforts [10]. Edentulism is significantly associated with disadvantaged socioeconomic status (SES) determinants [11, 12], edentulism, being the cause and consequence of social inequality, represents oral healthcare services, social, and economic burden, being the source and consequence of social inequality [13].

The life course epidemiology theory proposes that social and economic exposures during the childhood developmental period [14], have long-term effects on health outcomes or disease risk in adulthood [15]. Indeed, childhood SES inequalities in oral health remained with age [16], and showed a long-lasting relationship with childhood SES and oral health outcomes [17]. Childhood SES was found to have long-term consequences on severe tooth loss [18]. Disadvantaged childhood SES was found to have an effect on dental caries [19], and poor financial situation of the family in childhood was associated with the prevalence of unsound teeth in adulthood [20]. Being closely associated to SES, oral diseases disproportionally affect poorer SES groups [3]. Several studies have revealed that oral health inequalities are directly influenced by SES determinants [21–23], including education [24], and occupation [24]. Childhood SES was assessed based on parental SES determinants, including parental education [25] and occupation [26], self-reported financial situation of the family [27],primary residence [28] and food availability [29]. Disadvantaged childhood SES was also found to be related to oral healthcare service access [30, 31], which may contribute to severe tooth loss [32].

Oral conditions disparity in childhood, is a lifelong condition that can be tracked across the life course [33], increasing evidence indicates a direct effect of childhood SES on oral health in adulthood after controlling for adult SES [34], as assessed by educational achievements [26]. It has been reported that children who grew up in disadvantaged SES families had poorer oral health, with a threefold increase in low versus high SES families, in adult periodontal disease and dental caries [35]. A recent study highlighted that the effect of SES inequalities on health outcomes is a significant contributor to inequalities in childhood health [36], and the mediating models suggested that childhood SES may influence adult health *via* education and occupational exposures [37].

Traditional regression models estimate independent direct effects, but there are methodological challenges to estimate direct and indirect effects of childhood SES without some degree of collinearity with oral health outcomes between intermediate confounding factors [38]. Structural equation modeling (SEM) is a statistical approach to estimate the direct and indirect pathways of multiple variables that combines confirmatory factor analysis (CFA) and multiple regression analysis [39], and depends on the outcome variable to decompose effects that go through a chain, and its application in oral health has been well documented [40].

### Conceptual Framework

**Fig. 1:**
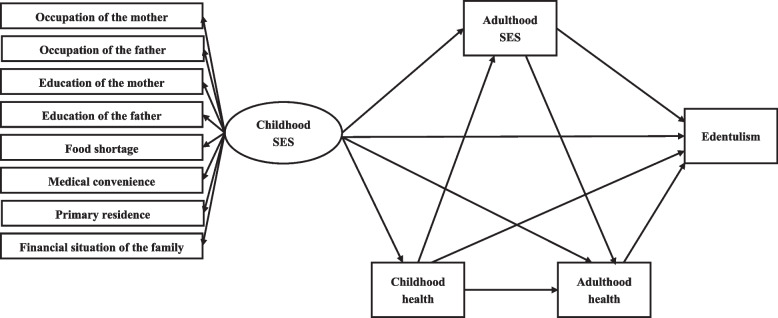
Conceptual model of the relationship between childhood SES and edentulism in mid-late adulthood. SES, socioeconomic status

A study has explored the association between childhood SES and edentulism in mid-to-late adulthood to understand the potential interventional policies using regression models [34]. Edentulism develops *via* complex pathways over the life course, but there is little evidence on the direct and indirect pathways from childhood SES and edentulism. According to the 2010 report “A Conceptual Framework for Action on the Social Determinants of Health” by the World Health Organization (WHO) [41], a context-specific database, and literature review, the core components of the conceptual framework in this study include: (1) Childhood SES associated with parents’ occupation and education, food shortage, medical convenience, primary residence, and financial situation of the family, which directly influence edentulism; (2) Childhood SES indirectly influence edentulism, *via* intermediate determinants including: adult SES, childhood health, and adult health. The multiple pathways from childhood SES to edentulism, as well as the statistical analysis strategy are shown in Fig. 1.

## Methods

### Study design and participants

This study used nationally representative data from the 2014 and 2015 waves of the China Health and Retirement Longitudinal Study (CHARLS) of Chinese residents aged 45 and above [42]. The CHARLS includes a four-stage, stratified, probability sampling procedure covering 10,803 households from 450 villages/residential communities in 28 provinces across mainland China [42]. The 2014 wave was a life history survey of 20,543 participants and retrospectively collected childhood SES [43]. The 2015 wave was a regular follow-up survey of 21,095 participants and collected information on adult SES and health outcomes in mid-late adulthood. This study matched the participants of the 2014 and 2015 waves according to their IDs to trace the information on childhood SES and health, adult SES and health, and the prevalence of edentulism. The minimum sample size for SEM with more than 7 constructs was 500 [44]. Participants aged under 45 years who had missing information on childhood SES and edentulism were excluded. After matching and exclusion, 17,032 participants were ultimately included in the current analysis. CHARLS was approved by the Ethical Review Committee of Peking University and informed consent was obtained from each participant at the time of participation [42].

### Measures

The prevalence of edentulism was determined based on the response to the question: “Have you lost all of your teeth?” and dichotomized into “Yes” or “No”.

Demographic characteristics included sex (male or female) and age groups (45–59, 60–69, 70–79, and 80 or above).

Childhood SES was assessed by eight variables: education of parents, occupation of parents, food shortage, financial situation of the family, primary residence and medical convenience [34, 43]. Education level of parents was categorized into “illiterate”, “primary school”, “middle school” or “high school and above”. Occupations of parents were categorized into “non-agricultural” or “agricultural”. Food shortage was assessed based on the question: “When you were a child before the age of 17 was there ever a time when your family did not have enough food to eat?” and dichotomized into “Yes” or “No” [45]. Financial situation of the family was assessed based on the question: “When you were a child before the age of 17, compared to the average family in the same community/village at that time, how was your family’s financial situation?” and rated as “a lot better off than them”, “somewhat better off than them”, “same as them”, “somewhat worse off than them” or “a lot worse off than them”. Primary residence was determined based on the question: “Where did you mainly live before you were 16 years old? was it in a village or a city/town? [rural or urban (city or town)]” and dichotomized into “urban” or “rural”. Medical convenience was determined based on the question: “Are you satisfied with the quality, cost, and convenience of local healthcare” and dichotomized into “Yes” or “No”.

Childhood health was based on the question: “how would you evaluate your childhood health, up to and including age 15?” and categorized into “excellent”, “very good”, “good”, “fair” and “poor”.

Adult SES was assessed by education [46]. Adult education was determined based on the question: “What’s the highest level of education you have attained now?”, this study used the categories of “illiterate”, “elementary school”, “middle school” or “high school and above”. Adult health was assessed by self-reported health. Self-reported health was based on the question: “how would you evaluate your adult health, after age 15?” and categorized into “excellent”, “very good”, “good”, “fair” and “poor”.

### Statistical analysis

Descriptive statistics, correlation analysis, and the calculations and estimates of the SEM were performed using the statistical software Stata 15.0 (Stata Corporation, College Station, TX, USA). No significant difference in the distribution of covariates was found between the final analysis sample and those with missing data in edentulism and childhood SES. Chi-squared (χ^2^) tests and Fisher’s Exact tests were performed to compare the categorical variables including childhood SES, childhood health, adult SES and adult health between participants with and without edentulism. Correlation analyses were performed to assess the correlation of childhood SES, childhood health, adult SES and adult health, with edentulism. On the basis of the conceptual framework, this study developed an analytical model using the SEM approach to evaluate direct and indirect effects of childhood SES on edentulism.

CFA was used to examine the hypothesized measurement model by evaluating the relationships among observed and latent variables [47]. The direct and indirect effects between outcome, observed and latent variables were determined by SEM. The total effect represents the sum of direct and indirect effects, mathematically expressed as follows [48]: c = c′ + ab, where c = total effect, c′ = direct effect, ab = indirect effect. Bias-corrected bootstrapping (2000 bootstrap samples) was then used to estimate the statistical significance of the direct and indirect effects of each pathway in the SEM. Multiple modification indices were used to adjust, modify and obtain the best-fit model [49], including the comparative fit index (CFI), incremental fit index (IFI), standardized root mean square residual (SRMR), and the root–mean–square error of approximation (RMSEA). The RMSEA and SRMR ≤ 0.06, CFI and IFI > 0.90 indicated an acceptable model [50]. Although χ^2^ values should be reported as one of the fit indices, they were highly sensitive to large sample sizes and thus were excluded [51]. *P* < 0.05 indicated statistical significance.

## Results

The results of descriptive statistics and univariate analysis are presented in Table 1. The average age of the participants was 63.74 ± 10.34 years, and 52% were females. Individuals whose parents’ occupation was agricultural were more likely to have edentulism than those whose parents’ occupation was non-agricultural (*p* < 0.05). Significant differences in the education level of the parents were determined between participants with and without edentulism (*p* < 0.05). Individuals with food shortage and medical inconvenience were more likely to have edentulism than those without food shortage and medical inconvenience during childhood (*p* < 0.05). Individuals whose primary residence was in a rural area were more likely to have edentulism than those whose primary residence was in an urban area (*p* < 0.05). Individuals who did not receive formal education were more likely to have edentulism than those with higher education level (*p* < 0.001). Individuals who self-reported poor health were more likely to have edentulism than those who did not self-report poor health (*p* < 0.01).

**Table 1:** Descriptive statistics of the participants with and without edentulism (*N* = 17,032)

Variables	Edentulism		N (%)	χ^2^	*P*-*value*
	Yes (*n* = 914), (%)	No (*n* = 16,118), (%)			
Sex				0.182	0.683
Male	432 (5.3)	7735 (94.7)	8167 (48.0)		
Female	482 (5.4)	8383 (94.6)	8865 (52.0)		
Age group				313.174	< 0.001
45–59	117 (1.8)	6522 (98.2)	6639 (41.8)		
60–69	287 (5.2)	5247 (94.8)	5534 (34.9)		
70–79	310 (8.9)	3171 (91.1)	3481 (21.9)		
≥ 80	31 (14.3)	186 (85.7)	217 (1.4)		
Marital status				110.497	< 0.001
Married	697 (4.7)	14,177 (95.3)	14,874 (87.3)		
Single	9 (6.6)	128 (93.4)	137 (0.8)		
Others	208 (10.3)	1813 (89.7)	2021 (11.9)		
**Childhood SES**					
Occupation of the mother				7.897	0.005
Agricultural	776 (5.4)	13,535 (94.6)	14,311 (93.3)		
Non-agricultural	35 (3.4)	996 (96.6)	1031 (6.7)		
Occupation of the father				13.395	< 0.001
Agricultural	727 (5.6)	12,346 (94.4)	10,799 (80.4)		
Non-agricultural	126 (3.9)	3064 (96.1)	3190 (19.6)		
Financial situation of the family				2.568	0.632
A lot worse	224 (5.7)	3726 (94.3)	3950 (23.2)		
Somewhat worse off	139 (5.2)	2553 (94.8)	2692 (15.8)		
Same	464 (5.3)	8258 (94.7)	8722 (51.3)		
Somewhat better	69 (4.8)	1369 (95.2)	1438 (8.5)		
A lot better off	13 (6.7)	181 (93.3)	194 (1.1)		
Education of the mother				47.321	< 0.001
No formal education	834 (5.9)	13,363 (94.1)	14,197 (86.2)		
Elementary school	47 (2.4)	1890 (97.6)	1937 (11.8)		
Middle school	4 (1.6)	247 (98.4)	251 (1.5)		
High school and above	3 (3.8)	75 (96.2)	78 (0.5)		
Education of the father				38.199	< 0.001
No formal education	574 (6.4)	8357 (93.6)	8931 (56.9)		
Elementary school	240 (4.3)	5368 (95.7)	5608 (35.7)		
Middle school	33 (3.8)	839 (96.2)	872 (5.6)		
High school and above	10 (3.5)	276 (96.5)	286 (1.8)		
Food shortage				12.490	< 0.001
Yes	661 (5.8)	10,759 (94.2)	11,420 (67.4)		
No	248 (4.5)	5283 (95.5)	5531 (32.6)		
Primary residence				9.267	0.003
Rural	794 (5.9)	12,584 (94.1)	13,378 (91.2)		
Urban	50 (3.9)	1242 (96.1)	1292 (8.8)		
Medical convenience				12.647	0.001
Yes	584 (4.6)	12,055 (95.4)	12,639 (91.0)		
No	86 (6.9)	1164 (93.1)	1250 (9.0)		
**Childhood health**				28.749	< 0.001
poor	76 (8.7)	793 (91.3)	869 (5.1)		
fair	68 (5.0)	1289 (95.0)	1357 (8.0)		
good	495 (5.6)	8298 (94.4)	8793 (51.6)		
very good	149 (4.7)	3018 (95.3)	3167 (18.6)		
excellent	126 (4.4)	2720 (95.6)	2846 (16.7)		
**Adulthood SES**					
Education				73.220	< 0.001
No formal education	288 (7.9)	3380 (92.1)	3668 (26.1)		
Elementary school	359 (5.8)	5821 (94.2)	6180 (43.9)		
Middle school	122 (3.9)	3009 (96.1)	3131 (22.3)		
High school and above	26 (2.4)	1059 (97.6)	1085 (7.7)		
**Adulthood health**					
Self-rated health				29.542	< 0.001
poor	177 (7.2)	2281 (92.8)	2458 (14.9)		
fair	304 (5.2)	5591 (94.8)	5895 (35.7)		
good	251 (4.9)	4907 (95.1)	5158 (31.2)		
very good	82 (4.3)	1831 (95.7)	1913 (11.6)		
excellent	41 (3.7)	1066 (96.3)	1107 (6.7)		

*SES *Socioeconomic status

The results of the correlation analysis between variables of childhood SES, childhood health, adult SES and, adult health, and edentulism are listed in Table 2. Edentulism was negatively correlated with urban residence (*r* = -0.025, *p* < 0.05), mother with non-agricultural occupation (*r* = -0.023, *p* < 0.05) and father with non-agricultural occupation (*r* = -0.029, *p* < 0.05), mother with higher education (*r* = -0.053, *p* < 0.05), father with higher education (*r* = -0.049, *p* < 0.05), family without food shortage (*r* = -0.027, *p* < 0.05), medical convenience (*r* = -0.030, *p* < 0.05), better childhood health (*r* = -0.030, *p* < 0.05), higher education in adulthood (*r* = -0.072, *p* < 0.05), and better health in adulthood (*r* = -0.037, *p* < 0.05).

**Table 2:** Correlation matrix of the variables (*N* = 17,032)

Variables	(1)	(2)	(3)	(4)	(5)	(6)	(7)	(8)	(9)	(10)	(11)	(12)
Occupation of the mother (1)	1.000											
Occupation of the father (2)	0.521*	1.000										
Financial situation of the family (3)	0.110*	0.182*	1.000									
Education of the mother (4)	0.230*	0.222*	0.119*	1.000								
Education of the father (5)	0.118*	0.255*	0.140*	0.363*	1.000							
Food shortage (6)	0.127*	0.134*	0.250*	0.148*	0.107*	1.000						
Primary residence (7)	0.655*	0.478*	0.106*	0.194*	0.117*	0.119*	1.000					
Medical convenience (8)	0.052*	0.065*	0.067*	0.067*	0.067*	0.058*	0.062*	1.000				
Childhood health (9)	0.015	0.048*	0.121*	0.025*	0.018*	0.058*	0.020*	0.041*	1.000			
Education (10)	0.182*	0.241*	0.141*	0.210*	0.207*	0.107*	0.220*	0.115*	0.061*	1.000		
Self-rated health (11)	0.058*	0.069*	0.100*	0.079*	0.059*	0.099*	0.060*	0.069*	0.120*	0.103*	1.000	
Edentulism (12)	-0.023*	-0.029*	-0.006	-0.053*	-0.049*	-0.027*	-0.025*	-0.030*	-0.030*	-0.072*	-0.037*	1.000

* *p* < 0.05

### SEM results

**Fig. 2:**
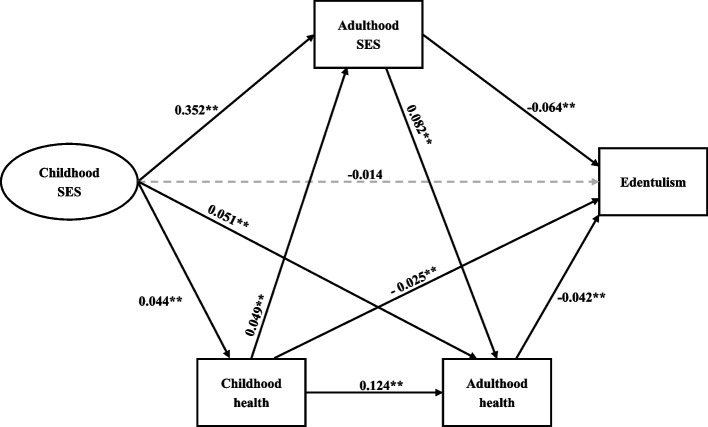
Structural equation model of the standardized pathways from childhood SES to edentulism in mid-late adulthood. Grey arrow refers to nonsignificant direct effects. Plain arrows depict significant direct effects. SES, socioeconomic status. ** *P*＜ 0.01. Fitting of the model: χ2/df = 2.34, RMSEA = 0.055; SRMR = 0.036; CFI = 0.924, IFI = 0.957

The standardized path estimates of the pathways from childhood SES to edentulism in late-life adulthood are shown in Fig. 2, and the standardized estimates of the direct, indirect and total effects of childhood SES on edentulism, as well as the specific effects *via* multiple pathways of childhood health, adult SES and adult health are shown in Tables 3 and 4. The standardized factor loadings of the CFA model of childhood SES were acceptable. Childhood SES had significant indirect (*β* = -0.026, *p* < 0.01), and total (*β* = -0.040, *p* < 0.05) effects on edentulism, but the direct effect of childhood SES on edentulism was insignificant (*β* = -0.014, *p* = 0.135). It was determined that 65% of the total effect of childhood SES on edentulism was indirect, and mainly mediated by adult SES. Specifically, childhood SES negatively predicted edentulism in late-life adulthood *via* poor health in childhood with an estimated indirect effect of -0.001 (*p* < 0.01). Childhood SES negatively predicted edentulism in mid-to-late adulthood *via* disadvantaged adult SES, with an estimated indirect effect of -0.023 (*p* < 0.01). Childhood SES negatively predicted edentulism in mid-to-late adulthood *via* poor adult health, with an estimated indirect effect of -0.001 (*p* < 0.01). Childhood SES significantly predicted edentulism in mid-to-late adulthood *via* the sequential mediation of childhood health and adult SES, with an estimated indirect effect of higher than 0.001 (*p* < 0.01). Childhood SES significantly predicted edentulism in mid-to-late adulthood *via* the sequential mediation of childhood health and adult health, with an estimated indirect effect higher than 0.001 (*p* < 0.01). Childhood SES significantly predicted edentulism in mid-to-late adulthood *via* the sequential mediation of childhood health, adult SES and adult health, with an estimated indirect effect higher than 0.001 (*p* < 0.01). The goodness-of-fit indices of the best-fitting model were acceptable, RMSEA and SRMR < 0.06, and CFI and IFI > 0.90.

**Table 3:** Standardized regression weight of variables in SEM model (*N* = 17,032)

Path Independent variable	Dependent variable	Standardized Estimate Parameter	S.E.	*P*-value
Childhood SES	Childhood health	0.044	0.009	< 0.001
Childhood SES	Adulthood SES	0.352	0.009	< 0.001
Childhood health	Adulthood SES	0.049	0.008	< 0.001
Childhood SES	Adulthood health	0.051	0.009	< 0.001
Childhood health	Adulthood health	0.124	0.008	< 0.001
Adulthood SES	Adulthood health	0.082	0.009	< 0.001
Childhood SES	Edentulism	-0.014	0.009	0.135
Childhood health	Edentulism	-0.025	0.008	0.001
Adulthood SES	Edentulism	-0.064	0.009	< 0.001
Adulthood health	Edentulism	-0.027	0.008	0.001

*SES *Socioeconomic status, *S.E *Standard error

**Table 4:** Standardized indirect, direct and total effects of childhood SES on edentulism (*N* = 17,032)

Pathways	Direct effects	Indirect effects	Total effects
From childhood SES to edentulism	-0.014	-0.026**	-0.040**
*Via* childhood health		-0.001**	
*Via* adulthood SES		-0.023**	
*Via* adulthood health		-0.001**	
*Via* childhood health and adulthood SES		> -0.001**	
*Via* childhood health and adulthood health		> -0.001**	
*Via* adulthood SES and adulthood health		> -0.001**	
*Via* childhood health and adulthood SES and adulthood health		> -0.001**	

*SES *Socioeconomic status. ** *P* < 0.01

## Discussion

To the best of our knowledge, this is the first study to explore the direct and indirect pathways from childhood SES to edentulism using a SEM approach in a LMIC. The findings of this study support the conceptual framework that the pathways from childhood SES to edentulism proceed *via* childhood health, adult SES, and adult health, which are targeted as oral health interventions to reduce the prevalence of edentulism and oral health inequities across the life course of individuals.

Due to the existence of multivariable and complicated mediators between childhood SES and edentulism, the SEM approach used in this study provided a clearer picture of the direct and indirect pathways from childhood SES to edentulism. This study identified an unexpected direct association between childhood SES and edentulism, which showed the importance of studying pathways from childhood SES to oral health outcomes. The findings of this study are consistent with those of previous life-course epidemiological studies [52, 53]. Two main chain risk models from childhood SES to edentulism were validated in this study: (1) Childhood SES was indirectly associated with edentulism mediated by childhood health; and (2) childhood SES was indirectly associated with edentulism mediated by adult SES.

Untreated dental caries in adulthood were negatively associated with childhood SES, both adults from LMICs and adults with disadvantaged SES in high-income countries (HICs) have life course history of oral disease [3]. Childhood SES affected tooth loss mediated by adult SES in Southern Brazil [54]. This study indicated that the indirect pathway from childhood SES to edentulism *via* adult SES. This is in agreement with previous study, in which the effect of disadvantaged childhood SES on oral health in adulthood was found to be mediated by adult SES [55]. SES has a cumulative effect on oral diseases, resulting in a pervasive SES over the life course [16]. Disadvantaged adult SES is associated with a higher risk of having dental lesions or dental experience [56], a severe dental caries [57], untreated dental caries lesions or lesion caries experience [56]. The findings in this study showed that SES has a cumulative effect on edentulism over the life course, thus highlighting the importance of childhood SES as an indirect effect on edentulism in adulthood. An advantaged early life is important to minimize the influence of chain risk and accumulative risk, but it is necessary to decrease SES inequalities by oral health recommendations during the whole life course in prevention of edentulism [4].

Accumulating evidence supports the suggestion that there is a relationship between oral and general health [58], which is agreement with the finding of this study, childhood SES significantly predicted edentulism in mid-to-late adulthood *via* the sequential mediation of childhood health and adult health. This is also in agreement with another previous study [59], supporting the accumulative risk model over the life course theory [54]. An earlier study also highlighted that edentulism-related health loss in childhood in LMICs was significantly associated with psychological challenges mediated by childhood health [60]. Swedish longitudinal surveys showed that financial hardship in childhood affects psychological distress in adulthood, and higher education in adulthood decreases the psychological distress [61]. The chain risk model is supported by the finding in this study indicating that childhood SES significantly indirectly predicted edentulism *via* the sequential mediation of childhood health and adult SES, the sequential mediation of childhood health and adult SES, and the sequential mediation of childhood health, adult SES and adult health.

### Recommendations for practice

Regardless of the childhood SES, the optimal oral health policy may overestimate the effects of other factors [34]. The current oral health policy lacks an analysis focused on pathways from childhood SES to oral health outcomes, and thus cannot completely tackle the global burden of oral diseases [62]. This study highlighted the urgent need to address childhood SES inequality as an oral health priority in LMICs [3]. A different strategy for the prevention of edentulism is needed to tackle the challenge, from a life course perspective, which considers how such an oral health SES policy can be implemented from childhood to mitigate the prevalence and development of edentulism in mid-to-late adulthood [4].

The implementation of intervention policies to address SES inequalities has been slow, the oral health community has been advocating the importance of integrated upstream and community based interventions, but oral healthcare services still operate as a non-integrated downstream oral program [4]. The prioritization of downstream interventions, such as clinical treatment services is an effective approach, that may decrease SES inequalities in oral health [63]. Oral policy makers rely on simplistic downstream interventions, due to the challenges of efficient evidence for the complex upstream interventions [3]. It is necessary to change the downstream interventions that cannot effectively achieve oral health gain or tackle inequalities, by creating resilient approaches for the prevention of upstream oral health [3]. Integrated and coordinated strategies targeting upstream prevention are required to tackle the underlying SES inequalities in oral diseases [4]. A reform of oral healthcare services is needed for cohesive, comprehensive, and integrated interventions, to address the neglect of oral health inequities in disadvantage childhood SES [4].

In LMICs access to dental prosthetic care may be significantly lower than in HICs [64].In China, in the past, tooth extraction was the main treatment for severe oral problems, which may be associated with edentulism [34]. In LMICs, the supply of medical resources, and investment on oral healthcare services are very limited, thus oral health practitioners may be insufficient, unavailable, and unaffordable for the public, particularly the disadvantaged SES population [4]. In China, due to the lack of access to primary and basic oral healthcare services for children and elders, such as hygiene guides, and oral health education, it is commercial pressures and treatment drives rather than oral health promotion and prevention [4]. The oral intervention approaches need to be effectively tailored to the needs of communities and individuals, to ensure oral health equity [4]. The distribution of medical care is a primitive outdated social form, and any return to it would exacerbate the maldistribution of medical resources [65]. Inequitable distribution of oral healthcare services may limit access to high quality and affordability of oral healthcare and preventive services, children with disadvantaged SES may become vulnerable to edentulism in mid-to-late adulthood, which could be avoided [34]. Households that pay the whole oral healthcare services have a higher risk of disadvantaged SES population, when spending a larger portion of disposable income [66]. It should be a priority to provide whole insurance coverage to support oral healthcare services for disadvantaged SES population from childhood [4].

### Strengths and limitations

This study has a number of limitations. First, edentulism was a self-reported outcome variable, which may potentially introduce reporting bias, although the self-reported oral health outcome was valid and reflected the real oral health status [67, 68]. Second, childhood SES data was represented by the parents’ SES were retrospective data, and although these variables were validated, memory bias cannot be ignored. To be considered for this, childhood SES was a latent variable, including education and occupation of the parents, food shortage, financial situation of the family, primary residence and medical convenience. Third, the prevalence of edentulism (5.8%) in this Chinese group was lower than that in the WHO Study on global AGEing and adult health (SAGE) (8.0–9.0%) [69]. The report of edentulism in the CHARLS database may suffer from survival bias. According to the CHARLS data, about 70% of participants did not report income in mid-to-late adulthood, the reason could be that 91.2% of them were living in rural areas, and did not receive a stable income. Therefore, this study did not use the variable of income to represent adult SES. Thus, this study was limited by the available variables and participants’ responses in the existing CHARLS database. Despite these limitations, this study has some strengths. For instance, this study used a unique and nationally representative data from a LMIC. The statistical analysis was conducted using SEM based on conceptual framework, which is an advanced statistical approach to estimate direct and indirect effects from exposure to outcome including latent variables. This is the first study using SEM to analyze the pathways from childhood SES to edentulism *via* childhood health, adult health and adult SES over the life course. This study is beneficial for addressing inequality in oral health from a life course perspective among the disadvantaged SES. 

## Conclusions

This study contributed to the evidence on the life course of the direct and indirect pathways from childhood SES to edentulism using SEM, and showed that childhood health, adult health and adult SES are mediators that explains this relationship in China. It suggested that it is necessary to explore integrated recommendations and intervention strategies from childhood to adulthood, considering the mediators of adult SES, childhood and adult health to mitigate the effects of childhood SES inequality on oral health outcomes. Currently, there is health inequality in access to oral healthcare services among the disadvantaged SES population that contributes to the inequality of oral health outcomes in LMICs. Primary oral healthcare services should consider that childhood health, adult health and adult SES may attenuate the effect of childhood SES inequality on the prevalence of edentulism in mid-to-late adulthood.

## Data Availability

Please contact CHARLS (China Health and Retirement Longitudinal Study) for data requests. http://charls.pku.edu.cn/zh-CN.

## Abbreviations

CFA: Confirmatory factor analysis
CFI: Comparative fit index
CHARLS: Chinese respondents in a Health and Retirement Longitudinal Study
DALYs: Disability-adjusted life years
GBD: Global Burden of Disease
HICs: High-income countries
IFI: Incremental fit index
LMICs: Low- and middle-income countries
RMSEA: the root–mean–square error of approximation
SAGE: Study on global AGEing and adult health
SEM: Structural equation modeling
SRMR: Standardized root mean square residual
SES: Socioeconomic status
WHO: World Health Organization

## References

[1] Kassebaum NJ, Smith AGC, Bernabe E, Fleming TD, Reynolds AE, Vos T, Murray CJL, Marcenes W, Collaborators GBDOH (2017). Global, Regional, and National Prevalence, incidence, and disability-adjusted life years for oral conditions for 195 countries, 1990–2015: a systematic analysis for the Global Burden of Diseases, Injuries, and risk factors. J Dent Res.

[2] Rousseau N, Steele J, May C, Exley C (2014). Your whole life is lived through your teeth’: biographical disruption and experiences of tooth loss and replacement. Sociol Health Illn.

[3] Peres MA, Macpherson LMD, Weyant RJ, Daly B, Venturelli R, Mathur MR, Listl S, Celeste RK, Guarnizo-Herreno CC, Kearns C (2019). Oral diseases: a global public health challenge. Lancet.

[4] Watt RG, Daly B, Allison P, Macpherson LMD, Venturelli R, Listl S, Weyant RJ, Mathur MR, Guarnizo-Herreno CC, Celeste RK (2019). Ending the neglect of global oral health: time for radical action. Lancet.

[5] Sgan-Cohen HD, Evans RW, Whelton H, Villena RS, MacDougall M, Williams DM, Steering I-G, Task G (2013). IADR global oral Health Inequalities Research Agenda (IADR-GOHIRA(R)): a call to action. J Dent Res.

[6] Nordenram G, Davidson T, Gynther G, Helgesson G, Hultin M, Jemt T, Lekholm U, Nilner K, Norlund A, Rohlin M (2013). Qualitative studies of patients’ perceptions of loss of teeth, the edentulous state and prosthetic rehabilitation: a systematic review with meta-synthesis. Acta Odontol Scand.

[7] Peltzer K, Hewlett S, Yawson AE, Moynihan P, Preet R, Wu F, Guo G, Arokiasamy P, Snodgrass JJ, Chatterji S (2014). Prevalence of loss of all teeth (edentulism) and associated factors in older adults in China, Ghana, India, Mexico, Russia and South Africa. Int J Environ Res Public Health.

[8] Emami E, de Souza RF, Kabawat M, Feine JS (2013). The impact of edentulism on oral and general health. Int J Dent.

[9] Thomson WM (2012). Monitoring edentulism in older New Zealand adults over two decades: a review and Commentary. Int J Dent.

[10] Matsuyama Y, Aida J, Tsuboya T, Hikichi H, Kondo K, Kawachi I, Osaka K (2017). Are lowered socioeconomic circumstances causally related to tooth loss? A natural experiment involving the 2011 Great East Japan Earthquake. Am J Epidemiol.

[11] Steele J, Shen J, Tsakos G, Fuller E, Morris S, Watt R, Guarnizo-Herreno C, Wildman J (2015). The interplay between socioeconomic inequalities and clinical oral health. J Dent Res.

[12] Peres MA, Barbato PR, Reis SC, Freitas CH, Antunes JL (2013). [Tooth loss in Brazil: analysis of the 2010 brazilian oral Health Survey]. Rev Saude Publica.

[13] Romandini M, Baima G, Antonoglou G, Bueno J, Figuero E, Sanz M (2021). Periodontitis, Edentulism, and risk of mortality: a systematic review with Meta-analyses. J Dent Res.

[14] Mishra GD, Kuh D, Ben-Shlomo Y (2003). Life Course Epidemiology. J Epidemiol Community Health (1979-).

[15] Ben-Shlomo Y, Kuh D (2002). A life course approach to chronic disease epidemiology: conceptual models, empirical challenges and interdisciplinary perspectives. Int J Epidemiol.

[16] Celeste RK, Fritzell J (2018). Do socioeconomic inequalities in pain, psychological distress and oral health increase or decrease over the life course? Evidence from Sweden over 43 years of follow-up. J Epidemiol Community Health.

[17] Listl S, Broadbent JM, Thomson WM, Stock C, Shen J, Steele J, Wildman J, Heilmann A, Watt RG, Tsakos G (2018). Childhood socioeconomic conditions and teeth in older adulthood: evidence from SHARE wave 5. Community Dent Oral Epidemiol.

[18] Fantin R, Delpierre C, Kelly-Irving M, Barboza Solis C (2018). Early socioeconomic conditions and severe tooth loss in middle-aged Costa ricans. Community Dent Oral Epidemiol.

[19] Peres MA, Peres KG, de Barros AJ, Victora CG (2007). The relation between family socioeconomic trajectories from childhood to adolescence and dental caries and associated oral behaviours. J Epidemiol Community Health.

[20] Peres MA, Peres KG, Thomson WM, Broadbent JM, Gigante DP, Horta BL (2011). The influence of family income trajectories from birth to adulthood on adult oral health: findings from the 1982 Pelotas birth cohort. Am J Public Health.

[21] Watt RG, Sheiham A (2012). Integrating the common risk factor approach into a social determinants framework. Community Dent Oral Epidemiol.

[22] Kim DW, Park JC, Rim TT, Jung UW, Kim CS, Donos N, Cha IH, Choi SH (2014). Socioeconomic disparities of periodontitis in Koreans based on the KNHANES IV. Oral Dis.

[23] Matsuyama Y, Aida J, Takeuchi K, Tsakos G, Watt RG, Kondo K, Osaka K (2014). Inequalities of dental prosthesis use under universal healthcare insurance. Community Dent Oral Epidemiol.

[24] Wennstrom A, Ahlqwist M, Stenman U, Bjorkelund C, Hakeberg M (2013). Trends in tooth loss in relation to socio-economic status among swedish women, aged 38 and 50 years: repeated cross-sectional surveys 1968–2004. BMC Oral Health.

[25] Setodeh S, Ghodrati F, Akbarzadeh M (2019). The efficacy of Father attachment education on the severity of domestic violence in Primegravida Women. J Caring Sci.

[26] Zhong Y, Wang J, Nicholas S (2017). Gender, childhood and adult socioeconomic inequalities in functional disability among chinese older adults. Int J Equity Health.

[27] Sheikh MA (2017). Childhood adversities and chronic conditions: examination of mediators, recall bias and age at diagnosis. Int J Public Health.

[28] Coffey D, Deshpande A, Hammer J, Spears D (2019). Local Social Inequality, Economic Inequality, and disparities in child height in India. Demography.

[29] Zhang Z, Gu D, Hayward MD (2008). Early life influences on cognitive impairment among Oldest Old Chinese. Journals of Gerontology.

[30] Babazono A, Kuwabara K, Hagihara A, Yamamoto E, Hillman A (2008). Does income influence demand for medical services despite Japan’s “Health Care for All” policy?. Int J Technol Assess Health Care.

[31] Arat A, Norredam M, Baum U, Jonsson SH, Gunlaugsson G, Wallby T (2020). Organisation of preventive child health services: Key to socio-economic equity in vaccine uptake?. Scand J Public Health.

[32] Tsakos G, Demakakos P, Breeze E, Watt RG (2011). Social gradients in oral health in older adults: findings from the English longitudinal survey of aging. Am J Public Health.

[33] Watt RG, Mathur MR, Aida J, Bonecker M, Venturelli R, Gansky SA (2018). Oral health disparities in children: a Canary in the Coalmine?. Pediatr Clin North Am.

[34] Zhang X, Chen S (2019). Association of childhood socioeconomic status with edentulism among chinese in mid-late adulthood. BMC Oral Health.

[35] Poulton R, Caspi A, Milne BJ, Thomson WM, Taylor A, Sears MR, Moffitt TE (2002). Association between children’s experience of socioeconomic disadvantage and adult health: a life-course study. Lancet.

[36] Ride J (2019). Is socioeconomic inequality in postnatal depression an early-life root of disadvantage for children?. Eur J Health Econ.

[37] Zhang Z, Gu D, Hayward MD (2008). Early life influences on cognitive impairment among oldest old chinese. Journals of Gerontology Series B-Psychological Sciences and Social Sciences.

[38] De Stavola BL, Daniel RM, Ploubidis GB, Micali N (2015). Mediation analysis with intermediate confounding: structural equation modeling viewed through the causal inference lens. Am J Epidemiol.

[39] Behbahanirad A, Joulaei H, Jamali J, Vossoughi M, Golkari A (2017). A model for oral health gradients in children: using structural equation modeling. Community Dent Health.

[40] Carmo CDS, Ribeiro MRC, Teixeira JXP, Alves CMC, Franco MM, Franca A, Benatti BB, Cunha-Cruz J, Ribeiro CCC (2018). Added Sugar Consumption and chronic oral disease burden among adolescents in Brazil. J Dent Res.

[41] A conceptual framework for action on the social determinants of health. Social Determinants of Health Discussion Paper 2 (Policy and Practice) [. https://www.who.int/sdhconference/resources/ConceptualframeworkforactiononSDH_eng.pdf]

[42] Zhao Y, Hu Y, Smith JP, Strauss J, Yang G (2012). Cohort Profile: the China Health and Retirement Longitudinal Study (CHARLS). Int J Epidemiol.

[43] Peele ME (2019). Domains of childhood disadvantage and functional limitation trajectories among midlife men and women in China. J Aging Health.

[44] Multivariate Data Analysis. https://is.muni.cz/el/1423/podzim2017/PSY028/um/_Hair_-_Multivariate_data_analysis_7th_revised.pdf.

[45] Tian F, Meng SS, Qiu P (2019). Childhood adversities and mid-late depressive symptoms over the life course: evidence from the China health and retirement longitudinal study. J Affect Disord.

[46] Qin Y, Zhang R, Yuan B, Xu T, Chen H, Yang Y (2019). Structural equation modelling for associated factors with dental caries among 3–5-year-old children: a cross-sectional study. BMC Oral Health.

[47] Karmakar M, Elhai JD, Amialchuk AA, Tietjen GE (2018). Do personality traits mediate the relationship between childhood abuse and migraine? An exploration of the Relationships in young adults using the add Health dataset. Headache: The Journal of Head and Face Pain.

[48] Vanderweele TJ (2015). Mediation analysis: a practitioner’s guide. Annu Rev Public Health.

[49] Browne M, Cudeck R (1992). Alternative ways of assessing model fit. Sociol Methods Res.

[50] Kline RB. Principles and practice of structural equation modeling; 2005. http://ndl.ethernet.edu.et/bitstream/123456789/74702/1/35.pdf.

[51] Schermelleh-Engel K, Moosbrugger H, Müller H (2003). Evaluating the fit of structural equation models: tests of significance and descriptive goodness-of-fit measures. Methods Psychol Res Online.

[52] Loucks EB, Lynch JW, Pilote L, Fuhrer R, Almeida ND, Richard H, Agha G, Murabito JM, Benjamin EJ (2009). Life-course socioeconomic position and incidence of coronary heart disease: the Framingham offspring study. Am J Epidemiol.

[53] Stringhini S, Batty GD, Bovet P, Shipley MJ, Marmot MG, Kumari M, Tabak AG, Kivimaki M (2013). Association of lifecourse socioeconomic status with chronic inflammation and type 2 diabetes risk: the Whitehall II prospective cohort study. PLoS Med.

[54] Vendrame E, Goulart MA, Hilgert JB, Hugo FN, Celeste RK (2018). Decomposing early and adult life social position effects on oral health and chronic diseases in a cross-sectional study of Southern Brazil. Community Dent Oral Epidemiol.

[55] Celeste RK, Eyjolfsdottir HS, Lennartsson C, Fritzell J (2020). Socioeconomic life course models and oral health: a longitudinal analysis. J Dent Res.

[56] Schwendicke F, Dorfer CE, Schlattmann P, Foster Page L, Thomson WM, Paris S (2015). Socioeconomic inequality and caries: a systematic review and meta-analysis. J Dent Res.

[57] Costa SM, Martins CC, Pinto MQC, Vasconcelos M, Abreu M (2018). Socioeconomic factors and caries in people between 19 and 60 years of age: an update of a systematic review and meta-analysis of observational studies. Int J Environ Res Public Health.

[58] Strait RH, Barnes S, Smith DK. Associations between oral health and general health: a survey wide association study of the NHANES. Community Dent Health. 2021;38(2):83–8. 10.1922/CDH_00121Strait06 34029020

[59] Zhang X, Jiang X, Sha M, Zhou Q, Li W, Guo Y, Ou Z, Cao J (2021). Life-course pathways from childhood socioeconomic status to type 2 diabetes in mid-late chinese adulthood. Sci Rep.

[60] Tyrovolas S, Koyanagi A, Panagiotakos DB, Haro JM, Kassebaum NJ, Chrepa V, Kotsakis GA (2016). Population prevalence of edentulism and its association with depression and self-rated health. Sci Rep.

[61] Darin-Mattsson A, Andel R, Celeste RK, Kareholt I (2018). Linking financial hardship throughout the life-course with psychological distress in old age: sensitive period, accumulation of risks, and chain of risks hypotheses. Soc Sci Med.

[62] Benzian H, Hobdell M, Holmgren C, Yee R, Monse B, Barnard JT, van Palenstein Helderman W (2011). Political priority of global oral health: an analysis of reasons for international neglect. Int Dent J.

[63] Qadri G, Alkilzy M, Franze M, Hoffmann W, Splieth C (2018). School-based oral health education increases caries inequalities. Community Dent Health.

[64] Gupta A, Felton DA, Jemt T, Koka S (2019). Rehabilitation of Edentulism and Mortality: a systematic review. J Prosthodont.

[65] Hart JT (1971). The inverse care law. Lancet.

[66] Bernabe E, Masood M, Vujicic M (2017). The impact of out-of-pocket payments for dental care on household finances in low and middle income countries. BMC Public Health.

[67] Matsui D, Yamamoto T, Nishigaki M, Miyatani F, Watanabe I, Koyama T, Ozaki E, Kuriyama N, Kanamura N, Watanabe Y (2016). Validity of self-reported number of teeth and oral health variables. BMC Oral Health.

[68] Jamieson LM, Thomson WM, McGee R (2004). An assessment of the validity and reliability of dental self-report items used in a National Child Nutrition Survey. Community Dent Oral Epidemiol.

[69] Kowal P, Chatterji S, Naidoo N, Biritwum R, Fan W, Lopez Ridaura R (2012). Data resource profile: the World Health Organization Study on global AGEing and adult health (SAGE). Int J Epidemiol.

